# Resonance assignments of the microtubule-binding domain of the microtubule-associated protein 7 (MAP7)

**DOI:** 10.1007/s12104-023-10124-8

**Published:** 2023-04-26

**Authors:** Agnes Adler, Lenette F. Kjaer, J. Wouter Beugelink, Marc Baldus, Hugo van Ingen

**Affiliations:** 1 Bijvoet Center for Biomolecular Research, NMR Spectroscopy, Utrecht University, Padualaan 8, Utrecht, 3584 CH The Netherlands; 2 Structural Biochemistry, Bijvoet Center for Biomolecular Research, Utrecht University, Padualaan 8, Utrecht, 3584 CH The Netherlands; 3 Institute of Structural Biology Grenoble, Grenoble, Auvergne-Rhône-Alpes, France

**Keywords:** NMR resonance assignments, Microtubule-associated proteins, Microtubules, MAP7, MTBD

## Abstract

The microtubule-associated protein 7 (MAP7) is a protein involved in cargo transport along microtubules (MTs) by interacting with kinesin-1 through the C-terminal kinesin-binding domain. Moreover, the protein is reported to stabilize MT, thereby playing a key role in axonal branch development. An important element for this latter function is the 112 amino-acid long N-terminal microtubule-binding domain (MTBD) of MAP7. Here we report NMR backbone and side-chain assignments that suggest a primarily alpha-helical secondary fold of this MTBD in solution. The MTBD contains a central long α-helical segment that includes a short four-residue ‘hinge’ sequence with decreased helicity and increased flexibility. Our data represent a first step towards analysing the complex interaction of MAP7 with MTs at an atomic level via NMR spectroscopy.

## Biological context

Microtubules (MT) represent a principal component of the cytoskeleton in eukaryotic cells. Besides other functions, they are essential in intracellular organisation, organelle trafficking and mitosis. The biopolymers are composed of αβ-tubulin heterodimers that assemble into hollow cylinders (Mandelkow et al. 1986). One MT characteristic that allows the processes guided by MT is their highly dynamic nature (Mitchison and Kirschner 1984). A misbalance in these processes can lead to diseases, including cancer, Alzheimer’s and Parkinson’s disease (Borys et al. 2020; Sferra et al. 2020).

The execution of the variety of MT tasks and the associated dynamics rely on the binding and interactions with so-called microtubule-associated proteins (MAPs) (Bodakuntla et al. 2019). While some MAPs have been studied to a great extent, others remain poorly characterised despite their essential functions (Goodson and Jonasson 2018).

**Fig. 1:**
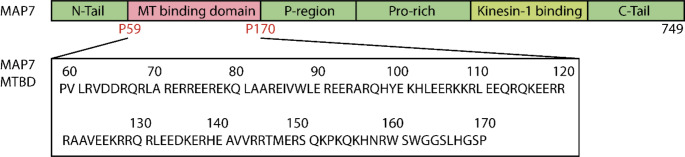
Domain organization **Top**: Schematic representation of full-length MAP7 including the MTBD and the kinesin-1 binding domain. **Bottom**: Amino-acid sequence of the MAP7 MTBD.

MAP7 (also known as E-MAP-115 or Ensconsin) is a MAP that is believed to activate the transport of kinesin-1 along the MT (Monroy et al. 2018; Métivier et al. 2018; Hooikaas et al. 2019). Furthermore, parallel MT sliding by Kinesin-1 might be promoted by MAP7. This function is regulated by its C-terminal kinesin binding domain (Metzger et al. 2012). Additionally, MAP7 is reported to stabilize and bundle MT with its N-terminal microtubule-binding domain (MTBD) (Fig. 1) (Sun et al. 2011; Kikuchi et al. 2018). This interaction seems to play an important role in regulating the MT dynamics during axonal branch development or metaphase spindle growth (Gallaud et al. 2014; Tymanskyj et al. 2017). In addition, MAP7 plays a role in motor-driven transport along the MT. In this role, its regulation is closely associated with that of the Alzheimer-associated protein Tau which also belongs to the class of MAPs. In contrast to the latter, Tau inhibits Kinesin-1 mobility (Ebneth et al. 1998; Dixit et al. 2008; Baas and Qiang 2019). Furthermore, Tau can replace MAP7 on MT, highlighting the complex coordination of the MT function and their associated proteins (Monroy et al. 2018).

MAP7 is thought to bind along the length of MT (Ferro et al. 2022). The cryogenic electron microscopy (cryo-EM) reconstruction from Ferro et al. 2022 revealed a 53 residue-long α-helix that binds a tubulin dimer of the MT protofilament between the outer ridge and the site of lateral contacts (PDBid:7SGS). Due to the challenges associated with the study of MAPs binding a longer stretch than a tubulin dimer by cryo-EM, this picture might underestimate the length of the binding region. Moreover, dynamic interactions between the two binding partners remain elusive with this method. Furthermore, the appearance of the MAP7 MTBD as a single α-helix is interesting, as sequence analysis using Waggawagga (Simm et al. 2015) shows high propensity for the formation of a coiled-coil but also indicates the MTBD could form a single alpha-helix (SAH) domain. Whether or not the MTBD alone is also a single α-helix in the free state is not known.

To allow for a more in-depth study of its structure and function, we have started an NMR study of MAP7 MTBD and its interaction with MTs. We adapted a purification protocol of the 112 residue MTBD construct (59–170) yielding pure protein and assigned its backbone and side-chains. The resulting chemical-shift assignments reveal that the MTBD free in solution contains a central long α-helical segment that includes a short four-residue ‘hinge’ sequence with decreased helicity and increased flexibility.

## Methods and experiments

The cDNA encoding the MTBD (residues 59–170) of Microtubule-associated protein 7 from *Homo sapiens* with an N-terminal His-Tag and Maltose binding protein (MBP) linked via a thrombin cleavage site (His-MBP-MAP7 MTBD), was cloned into the pLICHIS vector.

The protein was expressed in *Escherichia coli Rosetta 2 (DE3)* cells and the bacteria were grown using M9 minimal media containing 0.5 g/L ^15^NH_4_Cl and 2 g/L U-^13^ C glucose containing 100 mg/L ampicillin and 35 mg/L chloramphenicol. The culture was induced at an OD_600_ of 0.6 with 0.3 mM IPTG and incubated at 37 °C for 4 h. The cells were spun down at 4000 × *g*, 4 °C for 20 min to harvest cells. Cell pellets were resuspended with Buffer A (50 mM NaPi, pH 8; 150 mM NaCl; 1 mM beta-ME; 20 mM imidazole) with Lysozyme and stored at -80 °C.

For protein purification, the cells were lysed by sonication on ice and subsequently, the cellular debris was removed by centrifugation at 40,000 × *g*, 4 °C for 30 min. Then, the His-MBP-MAP7 MTBD was purified by HiTrap immobilized metal affinity chromatography (IMAC, (GE Healthcare Life Sciences)) (equilibrated with buffer A) attached to an ÄKTA Pure system. Afterwards the cell lysate was loaded onto the column and a wash with 20 column volumes (CV) with buffer A was performed before eluting the protein with buffer B (buffer A with 400 mM imidazole) supplemented with protease inhibitors (Sigma-Aldrich, cOmplete EDTA-free). The protein was concentrated in the presence of protease inhibitor using a 10 kDa molecular weight cut off Amicon filter (Sigma-Aldrich) and then diluted with cation exchange (CEX) buffer A (40 mM NaPi, pH 6.5) to a final concentration of 50 mM NaCl. Next, CEX was performed with the HiTrap HP SP chromatography column (GE Healthcare Life Sciences). The sample was loaded onto the column preequilibrated with CEX buffer A and washed with 10 CV CEX buffer A. The protein was eluted by applying a gradient to 100% CEX buffer B (40 mM sodium phosphate buffer, 1 M NaCl, pH 6.5). The MBP-MAP7 MTBD fusion protein was concentrated to a volume less than 1 mL and the His-MBP-tag was cleaved (leaving an additional two residues GS N-terminal to P59) overnight with 10 units of thrombin during dialysis with NMR buffer (40 mM phosphate buffer, 150 mM NaCl, 1 mM dithiothreitol (DTT), pH 6.5) to remove the high salt content. The His-MBP tag was removed by selective ammonium sulphate precipitation of MAP7 MTBD. In a first step 50% (NH_4_)_2_SO_4_ saturation was used to precipitate the protein. After the ammonium sulphate addition, the solution was mixed under inversion at 4˚C for 30 min. Subsequently, the solution was centrifuged at 10,000 xg, 15 min, 4˚C. The MAP7-MTBD pellet was washed with 40% (NH_4_)_2_SO_4_ saturation in NMR buffer by mixing by inversion at 4˚C for 30 min followed by centrifugation as previously. The pellet was resuspended in NMR buffer and buffer exchanged to NMR buffer overnight to remove remaining ammonium sulfate. The aggregation state of the protein was assessed using SEC-MALS by injecting 5 concentrations ranging from 0.2 to 3.2 mg/mL of purified Map7 MTBD on a Superdex 75 Increase column (Cytiva) calibrated with NMR buffer. A miniDAWN TREOS detector (Wyatt) and a RID-10 A differential refractive index monitor (Shimadzu) were used. Collected data was analyzed using ASTRA6 software (Wyatt).

For solution-state NMR measurements, a 150 µM sample of U-^15^ N,^13^ C-labeled MAP7 MTBD in NMR buffer supplemented with 5% D_2_O was used to acquire NMR spectra at 298 K on a Bruker Avance III HD 600 spectrometer or on a Bruker Avance III HD 900 instrument equipped with triple resonance cryogenic-probes. Backbone and side-chain resonance assignments were derived from ^1^ H to ^15^ N TROSY, ^1^ H–^13^ C constant time HSQC, HNCA, HNCACB, CBCA(CO)NH, HNCO, HN(CA)CO, HBHA(CO)NH, (H)C(CO)NH, CCH-TOCSY experiments. All spectra were processed using the Bruker TopSpin 3.6.2 software. Chemical shifts were referenced via the water resonance. Typical processing parameters were utilized, with apodization with cosine-squared window functions in all dimensions and doubling of the time domain signal by linear prediction in the indirect ^15^ N and ^13^ C dimensions. The spectra were analysed using POKY from NMRFAM-Sparky (Lee et al. 2021). The secondary structure (or the ϕ and ψ backbone torsion angles) predictions of the MAP7 MTBD were carried out using TALOS-N (Shen and Bax 2015) based on the chemical shifts of backbone resonances.

## Extent of assignments and data deposition

**Fig. 2:**
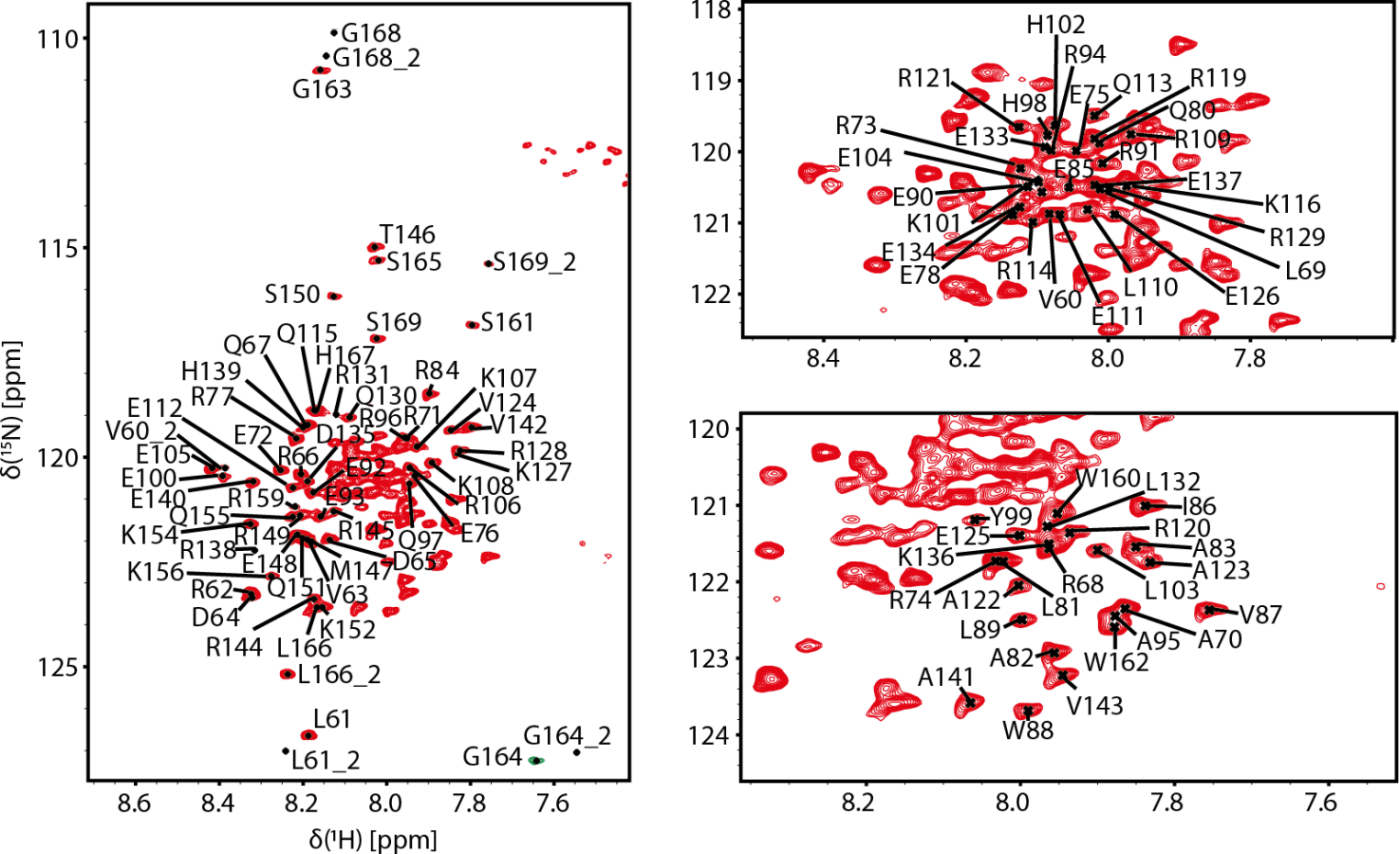
Resonance assignment of MAP7 MTBD. Two-dimensional ^15^ N-TROSY-HSQC of ^13^ C-^15^ N-MAP7 MTBD at 600 MHz acquired at 298 K. Reported amino acids are numbered according to the native sequence of the full-length protein. Second assignments of amino acids are denoted with a 2. Residues G164 and G164_2 are folded and displayed with green (negative) contour lines.

The MAP7 MTBD (residues 59–170, with an additional N-terminal GS thrombin cleavage scar) contains 114 amino acids and has a molecular weight of 14.23 kDa. Due to its high content of arginine (22.3%), glutamate (19.6%), lysine (8.9%) and glutamine (7.1%) combined with a high alpha-helical content, the ^1^ H-^15^ N TROSY spectrum is crowded (Fig. 2). The appearance of the line shapes suggests the MTBD may partly aggregate. Indeed, analysis of MAP7 MTBD with size-exclusion chromatography coupled with multi-angle light scattering (SEC-MALS) showed that the MTDB tends to oligomerize and indicated the protein is predominantly in a monomeric state at the concentration used for NMR (data not shown).

Despite severe overlaps, we were able to obtain near-complete backbone assignments: ^1^H_N_ (95%: 104 out of 109 non-proline residues), ^15^ N (95%: 104 out of 109 non-proline residues), ^13^Cα (97%: 109 out of 112), ^13^Cβ (93%: 104 out of 112), ^13^CO (93%; 104 out of 112), ^1^Hα (66%: 74 out of 112) and ^1^Hβ (63%: 71 out of 112). The unassigned non-proline residues were K79, E117, E118 due to the repetitive nature of the sequence and H157 and N158 because of line broadening. The chemical shift assignments (^1^ H, ^15^ N, ^13^ C) have been deposited in the BioMagResBank (http://www.bmrb.wisc.edu) under accession number 51,730. Interestingly, residues 59–61 of the extreme N-terminus and 164–169 of the extreme C-terminus gave rise to duplicate resonances, indicative of a second conformational state populated to ~ 30% based on the average signal-to-noise ratios of the duplicated resonances. This might be due to proline cis-trans conformations of proline 59 and proline 170. For P59 this is supported by the Cβ resonance chemical shifts, with 32 ppm for the major and 34.81 ppm for the minor state, which is in line with a trans and cis configuration, respectively (Schubert et al. 2002). For P170 the Cβ chemical shift is unfortunately not assigned.

**Fig. 3:**
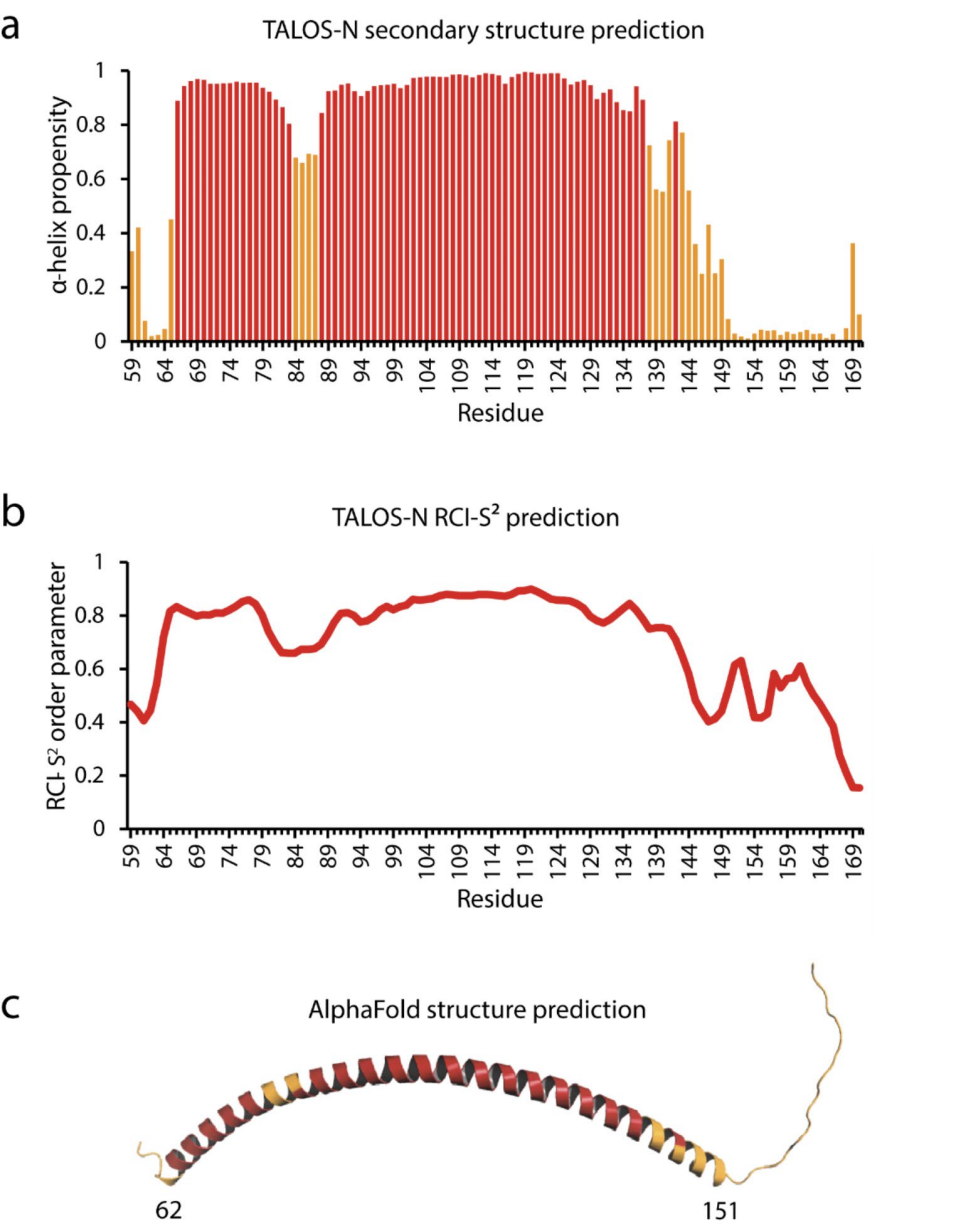
Predicted secondary structure using TALOS-N of MAP7 MTBD **(A)** TALOS-N secondary structure prediction with α-helix propensities higher than 0.8 colour-coded in red and lower than 0.8 in yellow. **(B)** Random coil index order parameters (RCI-S^2^) predicted by TALOS-N **(C)** The AlphaFold structure prediction is showing an α-helix ranging from residue 62 up to residue 151. Colour-coded in yellow are TALOS-N α-helix propensities lower than 0.8.

The secondary structure of MAP7 MTBD was predicted from the chemical shifts (^13^C_α_, ^13^CO, ^1^H_α_, amide ^1^HN and ^15^ N) using the TALOS-N program (Shen and Bax 2015). The protein forms an extended alpha-helical structure, spanning from residues 66 to 144 (α-helix propensity > 0.6), with disordered N- and C-termini (Fig. 3a). Residues 84 to 87 in the helical part have lower helical propensities and lower values for the random-coil-index-derived order-parameters (RCI-S^2^) (Fig. 3b). This indicates that MAP7 MTBD in its free state does not form a single stable helix as suggested from the AlphaFold prediction (Jumper et al. 2021) (Fig. 3c), but rather includes a more flexible internal ‘hinge’ sequence. In addition, the C-terminal end of the helix is ill-defined with decreasing helicity and increasing flexibility observed for residues 137–149. In contrast, the helix is sharply defined at the N-terminus, starting at residue 66, where the helix is stabilized by a *hpp*-*xpxhx* helix-capping motif (Aurora and Rosee 1998) formed by residues V63 to residue A70 (VDDRQRLA). In this motif, *h*/*p* denotes a hydrophobic/polar residue, *x* is any residue and the - marks the helix boundary.

## Summary

We here reported the chemical-shift assignments and secondary structure of MAP7 MTBD in its free state in solution. MAP7 MTBD adopts a predominantly α-helical secondary structure, with a central long α-helical segment from residue 66 to 144 and including a short ‘hinge’ from residue 84 to 87 with decreased helicity and increased flexibility.

Interestingly, the recent cryo-EM structure of MAP7 MTBD bound to microtubules suggested that the MTBD binds via a helix spanning at least residue 83 to 134 (Ferro et al. 2022). As we observed longer helical regions free in solution, both at the N- and C-terminus, this may indicate that the MTBD binding interface to microtubules is significantly more extended. Previously, solid-state NMR (ssNMR) has shown its potential to study MAP-MT interactions (see, e.g. (Atherton et al. 2017; Luo et al. 2020)) providing a strong incentive to study MAP7-MT complexes by ssNMR. This study hence represents a first step towards studying the MAP7 MTBD interaction with MT by ssNMR.

## Data Availability

Assignment deposited at the BioMagResBank under accession number 51,730.
